# Health-related quality of life in older adults: Effects of hearing loss and common chronic conditions

**DOI:** 10.12715/har.2015.4.4

**Published:** 2015-02-03

**Authors:** Annie N. Simpson, Kit N. Simpson, Judy R. Dubno

**Affiliations:** 1 Department of Healthcare Leadership and Management, Medical University of South Carolina, Charleston, SC, USA; 2 Department of Otolaryngology-Head and Neck Surgery, Medical University of South Carolina, Charleston, SC, USA

## Abstract

**Background:**

Recent findings on hearing loss (HL) and healthy aging have highlighted important clinical and policy implications concerning quality of life in older adults. Our objective was to explore the impact of HL on quality of life in older Americans, independent of survival, using the 2000 Medical Expenditure Panel Survey (MEPS) and 2010 Census data.

**Methods:**

A retrospective cohort of 2,567 subjects aged 60–90 in the MEPS who provided information on self-reported HL, health-related quality of life and chronic conditions. The EQ-5D visual analog scale (VAS) transformation was used to estimate marginal utility decrements for 5-year age categories and conditions, including HL, hypertension, diabetes, angina, joint pain, asthma, emphysema, or blindness. The modeled decrements were applied to the 2010 US census population to estimate annual quality-adjusted life years (QALYs) lost.

**Results:**

Of the respondents, 15.4% had mild HL and 1.1% had moderate/severe hearing loss. Other conditions (utility decrement) included: joint pain 53% (.0643), hypertension 47.2% (.0292), diabetes 15.6% (.0577), angina 9.8% (.0352), asthma 7.9% (.0288), emphysema 4.5% (.1186), blindness 0.8% (.0836), and average age 71.0 with decrements .0033 per year. The decrement from hearing loss ranked 4th at 174,689 in the US population.

**Conclusions:**

The substantial impact of hearing loss on healthy aging may not be obvious when quality of life decrements include survival or when diluted with younger populations. Careful consideration of clinical interventions for age-related HL is warranted and further research is needed on the effect of HL on quality of life in otherwise healthy older adults.

## Introduction

Patient-centered outcomes research (PCOR) is an important US policy initiative that is aimed at “promoting science that sees through the eyes of patients” [1]. The PCOR initiative is a timely response to the expected high medical care demand that the “silver tsunami” of ageing “baby boomers” will be placing on the health systems in developed countries [2]. Healthy aging is important for the “boomer” generation [3], and health policy makers and clinicians must understand the values that this population places on diseases and impairments to make the best patient-centered policy decisions. Healthy aging is a multifactorial construct that encompasses quality of life, social participation, minimum cognitive or functional impairment, and autonomy in activities of daily living [4]; all factors that are valued by baby boomers. The measurement of the global burden of disease due to specific illnesses is an important tool for informing public health policy choices [5]. However, general population burden of disease (BOD) estimates may be inadequate for formulating health policy for subpopulations, such as today’s baby boomers. This report uses hearing loss (HL) as an example of ranking diseases by BOD estimates for a subpopulation compared to the usually applied ranking for the total population.

Presbyacusis, or age-related HL, is among the most common chronic conditions of aging, with 26.7 million individuals age 50 and older reporting hearing loss; however, only approximately 14% of older adults use hearing aids [6]. These reports call attention to HL as a public health problem and emphasize the need to consider HL interventions [7]. To understand the effects of HL as a condition that deserves attention, the impact of patients’ health-related quality of life (HRQoL) should be assessed and compared to HRQoL decrements reported for other chronic conditions in the same population. At a time when cost control and cost-effective use of scarce health care resources is the focus of most policy makers, the understanding of how a condition affects patient-reported outcomes is the first step required to assess the value of the required health care services.

HL can affect many aspects of medical care and may be correlated with other health problems. Hearing loss has been associated with perceived problems in access to medical care [8], negative life events such as incident dementia [9], higher rate of falls [10], and poorer cognitive functioning [11]. However, age-related hearing loss is not a single disorder, but the accumulated effects of many factors, such as excessive ototoxic drug use or noise exposure, ageing, and disease-related effects associated with vascular conditions and dementia [9, 12]. Thus, HL is recognized as affecting many aspects of patients’ health, but its intrinsic effect on patients’ HRQoL is not well understood. Comparisons of the magnitude of the effect of HL to HRQoL decrements associated with common chronic illnesses and conditions of ageing were reported for a Finnish population. Saarni and colleagues showed that self-reported HL conferred a decrement in HRQoL similar to diagnoses of migraine, cataracts, glaucoma, psoriasis, or disturbing allergies in a population of 8,028 individuals aged 30 years and older [13]. However, the magnitude varied with the HRQoL instrument used. To understand the scope of the impact of HL on HRQoL of older adults in the United States (US), we estimated the decrements associated with self-reported HL using national survey data in which HRQoL was measured using the EQ-5D visual analog scale (VAS) [14]. We then converted the global HRQoL measure to a calculated utility score using published conversion approaches [15] and ranked the impact of common chronic conditions, including HL, on total HRQoL for the US population.

## Methods

### Study population

This study examined data for 2,567 subjects, ages 60–90, in the 2000 Medical Expenditure Panel Survey (MEPS). Self-reported HL, HRQoL, and chronic conditions present were extracted from the publicly available 2000 national MEPS survey. The MEPS sample was limited to individuals 60–90 years of age in order to represent the aging population. HL was defined as “mild” if the respondent indicated they “can hear most conversations”, and “moderate/severe” if they could hear “some conversations”. The EQ-5D VAS response was used as the indicator of HRQoL [16]. We compared scores from patients with “mild” and “moderate/severe” HL to scores for patients who reported having no HL but were told by a health professional that they have one or more of the following conditions: hypertension, diabetes, coronary artery disease, joint pain, stroke, arthritis, asthma, depression, emphysema or chronic obstructive pulmonary disease (COPD), or blindness. Visual analog scale scores were converted to utilities for standardization and direct comparison.

The MEPS data sample was extracted from a public use data set that contains no subject identifiers, and was therefore classified as non-human research.

### Statistical analysis

**Figure 1:**
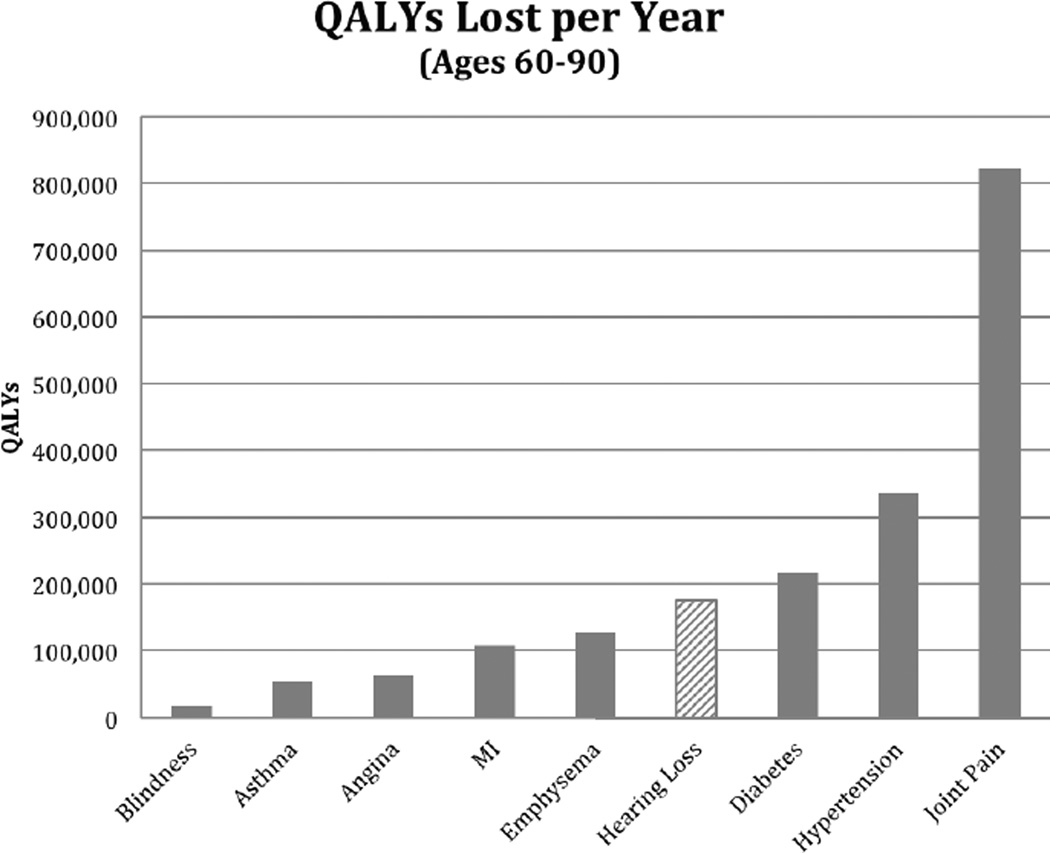
Total (quality-adjusted life year (QALY)) in one year (2010 US Population, ages 60–90)

Age, gender, and race distributions were examined using means and proportions, as appropriate. The EQ-5D VAS responses in the MEPS data were converted to utilities using an approach developed by Dolan [15] and applied to a US population by Luo and colleagues [17]. These measures of HRQoL were used to compare the association between HL and several other chronic conditions using multivariable regression models that additionally adjusted for age, gender, and race when these were statistically significant. Multivariable regression models were used to examine the relative contribution of HL and the reported medical conditions to the quality of life scores. We used the results from the MEPS sample to estimate the effects of the decrements associated with each condition for the total US population age 60–90 year in 2010. This population estimate indicates the number of quality-adjusted life years (QALYs) lost due to each chronic condition. We used 2010 age-specific population counts reported by the US census [18] multiplied by the percent of MEPS patients reporting each condition. We then assigned the utility decrements from the multivariable regression MEPS utility model, while controlling for the effects of age using five-year age categories. Decrements in total QALYs for the 2010 population for each health condition were estimated using the MEPS EQ-5D model results (Fig. 1).

All statistical analyses were conducted using SAS version 9.3 statistical software (Cary, NC). Population estimates were performed using Microsoft Excel.

## Results

Respondents in the MEPS national survey data set had a mean age of 71.0 years (range 60–90, SD 7.8); 45.7% were male, and 10.5% of black race; 15.4% reported mild HL and 1.0% reported moderate/severe hearing loss; 49.0% reported a diagnosis of one or more of: myocardial infarction, asthma, angina, diabetes, joint pain, emphysema, cancer, hypertension, kidney disease, osteoporosis, stroke, blindness, or memory problems. The mean EQ-5D VAS scores observed were 72.6 (range 1–100, SD 19.0) and the mean utility score based on the reported VAS score was 0.81 (range 0.49–0.93, SD 0.08), which is representative of the US population over the age of 65 [17]. Subjects’ descriptive characteristics are summarized in Table 1.

Moderate-to-severe HL (EQ-5D Utility −0.079, p<0.001) was associated with decrements double that of blindness (EQ-5D Utility −0.037, p=0.03) and 30% worse than emphysema (EQ-5D Utility −0.052, p<0.0001). Health-related quality of life utilities and p-values comparing quality of life decrements due to HL and other health conditions are shown in Table 2.

Applying the utility loss for a condition to the total US population integrates the effects of prevalence of a condition with the utility decrement associated with the specific disease. The greatest number of QALYs lost in 2010 for the US population over the age of 60 is due to the effects of diabetes, hypertension, and joint pain. However, approximately 175,000 QALYs were lost due to HL (Fig. 1), which surpasses losses from conditions such as emphysema, asthma, and angina (Fig. 1).

## Discussion

These results add to a growing body of evidence that HL significantly affects patients’ evaluation of their quality of life. Mild HL appears to have a small effect on HRQoL, as indicated by a utility score of −0.01, indicating a 1% decrement in HRQoL (Table 2). For the EQ-5D VAS the effect of HL on HRQoL is slightly less than angina, but slightly more than hypertension or asthma. Moderate/severe HL conferred a clear decrement of HRQoL, with an effect size similar to emphysema or blindness.

These exploratory findings warrant careful consideration of age-related HL in clinical practice and further research to examine the effect of impaired hearing and of the need for hearing loss interventions. Significant differences in some models related to age, gender, and race emphasize the importance of providing rehabilitation approaches that can be adapted to patient needs and preferences, and adjusted according to the etiologies of age-related HL. Given that a large proportion of patients with HL do not use hearing aids, our findings that HL is clearly associated with decrements in HRQoL on par with common chronic conditions make it clear that a pervasive void in understanding the need for intervention may exist, both among patients and medical practitioners. HL may be expected to have a substantial impact on healthy aging, which is not obvious when quality of life decrements are calculated to include survival, or when its effects are diluted by including younger populations as is commonly done in studies of national and international burden of illness. From a public health perspective HL ranks 31 in the global burden of disease (GBD) [11, 19], with much of the decrement being conferred by HL in developing countries. In contrast, HL ranks 4^th^ when estimating the impact on healthy aging in the US geriatric population. It is important that we begin to examine ways to close this knowledge gap before the large cohort of aging baby boomers begin to experience the loss of HRQoL that is evident with presbyacusis.

## Figures and Tables

**Table 1:** Characteristics of the Medical Expenditure Panel Survey (MEPS) sample

	MEPS (N=2,567)
Age M (SD)	71.0 (7.8)
	Range 60–90
	EQ-5D VAS Utility
HRQoL Score M (SD)	0.81 (0.08)
	Range 0.49–0.93
	N (%)
Male	1,172 (45.7%)
Black	270 (10.5%)
One or more chronic condition	6,146 (49%)
Hearing Loss	
No HL	2,146 (83.6%)
Mild HL	395 (15.4%)
Moderate/severe HL	26 (1.0%)

HL: hearing loss; HRQol: health-related quality of life

**Table 2:** Health-related quality of life (HRQoL) utilities for incremental levels of hearing loss (HL)

MEPS Data Variable Name	EQ-5D VAS Utility Scores (N=2,567)	p-value
Intercept	0.946	
Mild HL	−0.015	<0.001
Mod./Sev. HL	−0.079	<0.0001
Angina	−0.016	0.01
MI	−0.020	<0.001
Diabetes	−0.025	<0.0001
Joint Pain	−0.028	<0.0001
Hypertension	−0.013	<0.0001
Asthma	−0.013	0.03
Emphysema	−0.052	<0.0001
Blind	−0.037	0.03

Note: Multivariable model controlled for age, gender, and race when statistically significant.

HL: hearing loss; MEPS: Medical Expenditure Panel Survey; MI: myocardial infarction; VAS: visual analog scale
